# Unfolding of viral protein 1 N-termini facilitates genome ejection from recombinant adeno-associated virus serotype 8

**DOI:** 10.1016/j.omtm.2025.101480

**Published:** 2025-04-24

**Authors:** Yuki Yamaguchi, Saki Shimojo, Tomohiko Ikeda, Mitsuko Fukuhara, Yasuo Tsunaka, Risa Shibuya, Mark Allen Vergara Rocafort, Ryoji Nakatsuka, Kiichi Hirohata, Tetsuo Torisu, Susumu Uchiyama

**Affiliations:** 1 Department of Biotechnology, Graduate School of Engineering, The University of Osaka, 2-1 Yamadaoka, Suita, Osaka 565-0871, Japan; 2 U-Medico Inc., 2-1 Yamadaoka, Suita, Osaka 565-0871, Japan

**Keywords:** gene therapy, adeno-associated virus, genome release, thermal unfolding, mass photometry, nano-differential scanning fluorimetry, hydrogen/deuterium exchange mass spectrometry, analytical ultracentrifugation

## Abstract

The role of viral protein (VP) 1 and VP2, which comprise the recombinant adeno-associated virus (rAAV) capsid, in heat-induced genome release was investigated using rAAV serotype 8 (rAAV8) samples with a high VP1/VP2 to VP3 ratio, a low VP1/VP2 to VP3 ratio, and VP3 only. The thermal unfolding of the VP1 N-termini was closely monitored by nano-differential scanning fluorimetry with an onset temperature (*T*_onset1_) of ∼55°C and a melting temperature of ∼60°C (which was below the onset temperature of capsid disassembly [*T*_onset2_] >70°C), which is related to genome release upon heating. The folded VP1 N-termini prevented release of the full-length genome at temperatures below 60°C, whereas unfolding of the VP1 N-termini facilitated genome release above 60°C. Above *T*_onset1_ and below *T*_onset2_, most rAAV8 particles remained as monomeric particles in three states: capsids encapsidating their single-stranded DNA (ssDNA), capsids that had fully released their genome, and capsids that had fully ejected the genome while tethering the genome on the capsid surface as evidenced by large frictional ratios in analytical ultracentrifugation. The ratio of VP1 and/or VP2 to total VPs had little effect on the extent of genome release. These findings provide new insights into heat-induced genome release from rAAV at the molecular level.

## Graphical abstract

**Figure:**
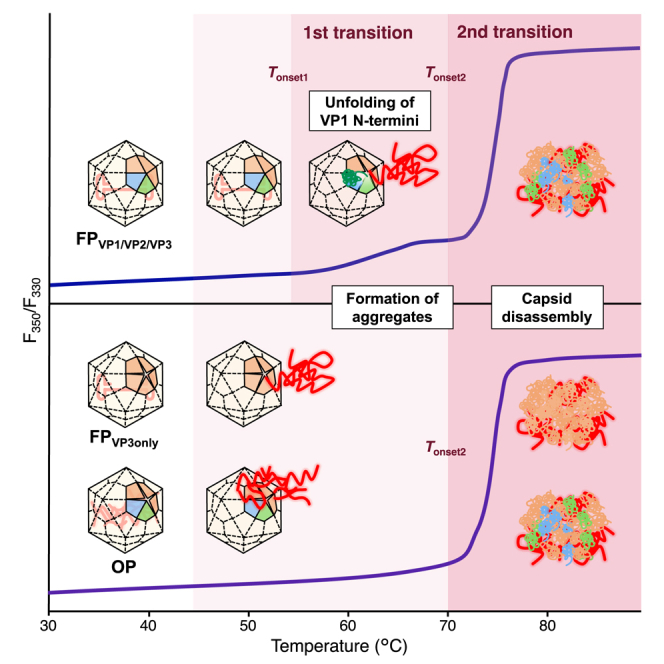

## Teaser

Uchiyama and colleagues closely monitored thermal unfolding of VP1 N-termini between 60°C and 70°C by nano-differential fluorimetry, which is related to genome release upon the heating; folded VP1 N-termini prevented the genome release below 60°C, whereas the unfolding of VP1 N-termini facilitated the genome release above 60°C.

## Introduction

Adeno-associated virus (AAV) is a nonenveloped virus that encapsidates single-stranded DNA (ssDNA) of approximately 4.7 kilobases (kb) in an icosahedral capsid.^1^ AAV belongs to the *Dependoparvovirus* genus of the Parvoviridae family, and requires coinfection with adenovirus as a helper virus for its replication. Recombinant AAV (rAAV) is a gene delivery vehicle for gene therapy.^2^ rAAV encapsidates the designed genome by replacing the *Rep* gene for viral replication and packaging and the *Cap* gene for viral protein (VP) expression, flanked by the two inverted terminal repeats in the wild-type genome, with a promoter and gene of interest to treat diseases.^2^ Its capsid consists of 60-mers of three major VPs—VP1, VP2, and VP3—in order of molecular weight. VP1 contains the VP1 unique (VP1u) region at the N-terminus that is not present in other VPs and possesses phospholipase A2 activity for endosomal escape.^3^ The VP2 N-terminus, which is shared with VP1, is designated the VP1/VP2 common region and contains a nuclear localization signal.^4^ Although the stoichiometric ratio of VPs that constitute the capsid has been considered to be VP1:VP2:VP3 = 5:5:50, recent studies have shown that rAAV is a mixture of heterogeneous particles with various VP stoichiometric ratios^5^^,^^6^ and that the incorporation of VP1 or VP1/VP2 into total VPs is highly correlated with rAAV transduction efficiency.^7^^,^^8^

Although some researchers have focused on the release of the rAAV genome, reports are contradictory. Atomic force microscopy observations of heat-induced rAAV genome release revealed that some rAAV particles release ssDNA while retaining almost intact capsids.^9^^,^^10^ By contrast, Ebberink et al. reported that upon DNA release, the capsids do not transform into empty rAAVs, but rather appear to aggregate or disintegrate.^11^ Two articles reported that the greater the size of the ssDNA genome, the more unstable the capsid and the greater the release of the rAAV genome at lower temperatures.^9^^,^^12^ By contrast, Barnes et al. reported that capsids encapsidating ssDNA of a similar size to the wild-type genome (approximately 4.7 kb) are the most stable, whereas capsids with shorter and longer genomes release their genomes at lower temperatures.^13^ Horowitz et al. reported that genome release occurs at lower temperatures at pH 7 than at pH 6 and at pH 6 than at pH 5,^9^ whereas Hsu et al. reported that at pH 4 and pH 5 the genome is extruded from the capsids more rapidly than at pH 6 and pH 7.^14^ Another study revealed that the increase in the redshift of tryptophan upon heating correlates with ssDNA accessibility of SYBR Gold; that is, the ssDNA was initially and partially released at the medium structural transition temperature range (55°C–60°C) and completely released at the major structural transition temperature range (64°C–70°C).^15^ Several factors may explain this discrepancy. One factor may be differences in the purity of the particles in the samples studied. For example, rAAV full particle (FP) samples should be composed solely of particles encapsidating designed ssDNA, whereas rAAV samples frequently contain partially filled particles that encapsidate DNA fragments and/or overpackaged particles (OPs) that encapsidate ssDNA longer than the designed ssDNA. Compared with FPs, such drug-related impurities may exhibit different genome release behaviors. The discrepancy could also be due to the VP stoichiometric ratio because we recently discovered that rAAVs sometimes have two FP fractions of different densities with different VP stoichiometric ratios, i.e., the ratio of VP1/VP2 to VP3.^6^^,^^8^ The same phenomenon may also appear differently because of the limitations of each analytical method.

Here, we focused on the VP stoichiometric ratio of rAAV serotype 8 (rAAV8) and investigated heat-induced genome release and capsid thermal stability using several biophysical methods, namely, mass photometry (MP), nano-differential scanning fluorimetry (nano-DSF), hydrogen/deuterium exchange mass spectrometry (HDX-MS), and sedimentation velocity analytical ultracentrifugation (SV-AUC). We prepared three rAAV8 samples with different VP stoichiometric ratios, including particles with a high VP1/VP2 to VP3 ratio, a low VP1/VP2 to VP3 ratio, and VP3 only. All of the samples were extensively purified to remove empty particles (EPs) with no encapsidated genome inside the capsid using cesium chloride density gradient ultracentrifugation (CsCl DG-UC). Moreover, a commercially available rAAV8 was fractionated, and genome release from the rAAV8 sample, which contains both FPs and OPs, was assessed. The results indicated that rAAV8 particles eject their genome from almost intact capsids upon heating, partly as monomeric particles and partly as aggregates, and that conformational changes in the VP1u and VP1/VP2 common regions (VP1 N-termini) occur before denaturation of the shared VP3 region and influence genome ejection. Furthermore, ssDNAs smaller than the designed size are released at temperatures lower than those required to unfold the VP1 N-termini. These results will help elucidate the uncoating process of the rAAV genome.

## Results

### Characterization of low-density, high-density, and VP3-only rAAV8 particles

**Figure 1:**
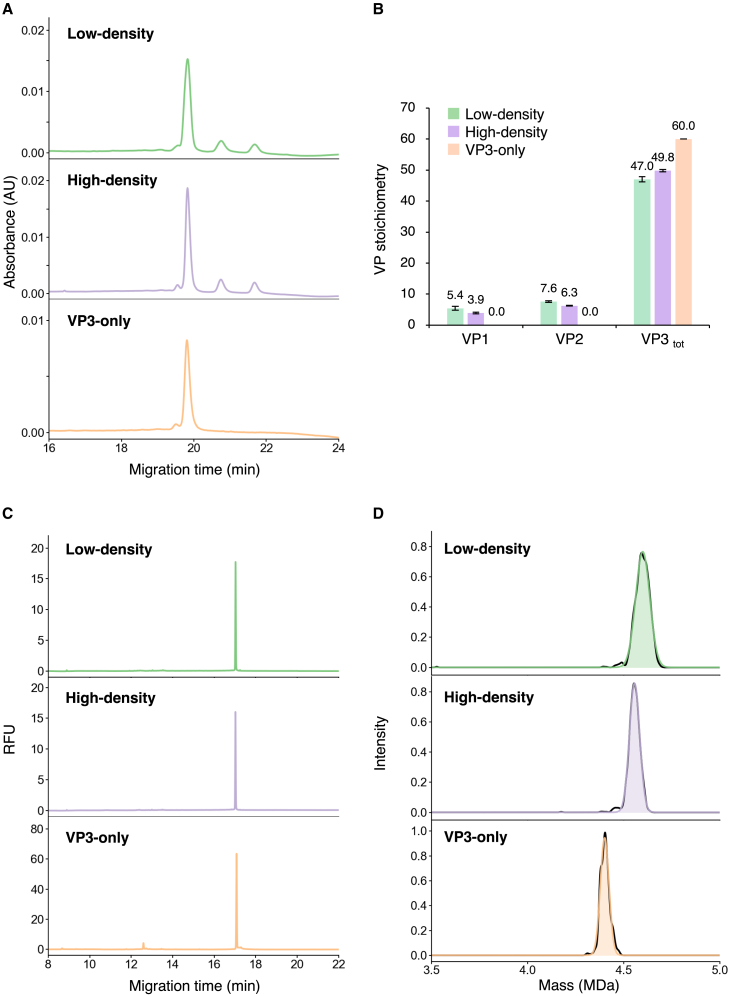
Characterization of rAAV8 particles in low-density and high-density fractions after two cycles of CsCl DG-UC and rAAV8 particles composed of VP3 only (A) CE-SDS electropherograms of VPs incorporated into the capsids for low-density, high-density, and VP3-only particles. (B) VP stoichiometric ratio for low-density, high-density, and VP3-only particles. Each error bar represents the standard deviation (SD) of triplicate measurements. (C) CE-LIF electropherograms of ssDNA encapsidated in low-density, high-density, and VP3-only particles. (D) Orbitrap-based CD-MS distributions for low-density, high-density, and VP3-only particles.

We prepared three rAAV8 samples, each with a different VP stoichiometric ratio: particles with a high ratio of VP1/VP2 to VP3, a low ratio of VP1/VP2 to VP3, and VP3 only. The buoyant densities of typical nucleic acids and proteins are 2.0 mg/cm^3^ and 1.4 mg/cm^3^, respectively.^16^ In our previous study, when the same length of genome was encapsidated in the capsid, rAAV2 with a high ratio of VP1/VP2 to VP3 had a lower buoyant density and a higher mass than rAAV2 with a low ratio of VP1/VP2 to VP3.^8^ After two cycles of CsCl DG-UC to purify the in-house rAAV8, low-density and high-density fractions were collected separately. Capillary gel electrophoresis using sodium dodecyl sulfate with UV detection (CE-SDS) revealed that the VP stoichiometric ratios for low-density and high-density particles were VP1:VP2:VP3_total_ (VP3 + VP3_variant_) = 5.4:7.6:47.0 (±0.6:0.2:0.8) and VP1:VP2:VP3_total_ = 3.9:6.3:49.8 (±0.3:0.1:0.4) respectively (Figures 1A and 1B). For VP3-only, in which the initial codons of both VP1 and VP2 were mutated, as expected, only peaks corresponding to VP3 and VP3_variant_ were observed (Figures 1A and 1B). Capillary gel electrophoresis with laser-induced fluorescence detection (CE-LIF) of samples pretreated with Benzonase showed major peaks at the same migration times for low-density, high-density, and VP3-only particles, indicating that all of the particles encapsidated the same length of ssDNA genome (Figure 1C). The length of the ssDNA for the main peak was 2,358 ± 3 bases for low-density particles, corresponding to the designed ssDNA of 2,526 bases. Orbitrap-based charge detection mass spectrometry (CD-MS) detected peaks at 4.61 ± 0.01 MDa, 4.56 ± 0.00 MDa, and 4.40 ± 0.00 MDa for the low-density, high-density, and VP3-only samples, respectively (Figure 1D). These peaks correspond to the FPs of 4.53 MDa, 4.49 MDa, and 4.36 MDa, for the respective samples, as calculated from the VP stoichiometry determined by CE-SDS and the theoretical length of encapsidated ssDNA. A small peak in the electropherogram was observed at 12.6 min for VP3-only, whereas no particles other than the FP peak, such as OPs, were detected suggesting that the FP peak contains a few particles encapsidating the full-length ssDNA genome and DNA fragments, which could not be resolved by our CD-MS method or were lower than the limit of detection, as described in our previous report.^17^ No peaks corresponding to EPs were detected in any of the samples. Thus, three types of rAAV8 particles with different VP stoichiometric ratios and the same ssDNA genome length were successfully prepared for the following experiments.

### Heat-induced genome release of low-density, high-density, and VP3-only rAAV8 particles

**Figure 2:**
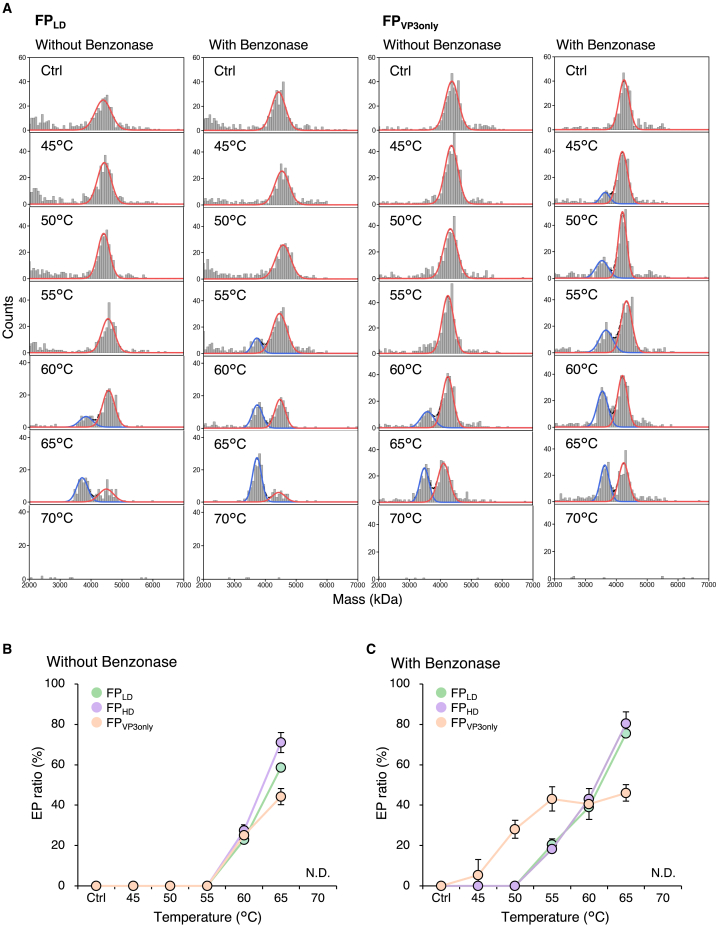
Observation of heat-induced genome release from rAAV8 FP_LD_, FP_HD_, and FP_VP3only_ (A) MP histograms of Benzonase-untreated and Benzonase-treated FP_LD_ and FP_VP3only_ after incubation on ice (control), and at 45°C, 50°C, 55°C, 60°C, 65°C, and 70°C. The blue and red lines represent areas of EPs and FPs, respectively. (B and C) EP ratio of (B) Benzonase-untreated and (C) Benzonase-treated FP_LD_, FP_HD_, and FP_VP3only_ after incubation on ice (control), and at 45°C, 50°C, 55°C, 60°C, 65°C, and 70°C. Each error bar represents the SD of triplicate measurements.

rAAV8 samples with different VP stoichiometric ratios were incubated on ice (control) and at 45°C, 50°C, 55°C, 60°C, 65°C, and 70°C for 15 min, after which their particle distributions were evaluated using MP (Figures 2A and S1). No peak corresponding to EPs was observed in the control samples for the three types of rAAV8, an observation that is supported by the results from the CD-MS measurements (Figure 1D). Therefore, in the following description, rAAV8 samples containing low-density particles are denoted as FP_LD_, high-density particles as FP_HD_, and VP3-only particles as FP_VP3only_. The proportion of EPs increased, whereas that of FPs decreased at higher incubation temperatures up to 65°C, and no particles were detected in the range of 2–7 MDa after incubation at 70°C. As explained in detail later, the same samples used for the MP analysis were subjected to nano-DSF to obtain thermodynamic parameters: onset temperature (*T*_onset_), melting temperature (*T*_m_), enthalpy change (Δ*H*), and entropy change (Δ*S*). We found that the *T*_onset_ of the second transition (*T*_onset2_), corresponding to the starting temperature of VP3 denaturation and capsid disassembly, was 71°C–72°C (Table 1), indicating that rAAV8 particles were not disassembled below 70°C. Therefore, there was little overlap between genome release and capsid disassembly below 70°C, and the lack of detection of particles after 70°C incubation was not a result of capsid disassembly, but presumably the formation of large aggregates mediated by the released genome. The possibility of overlapping genome release and capsid disassembly at incubation temperatures above 71°C–72°C cannot be excluded. In the absence of Benzonase treatment, the percentage of EPs did not increase in all three types of rAAV8 samples up to 60°C and increased only when the samples were heated above 60°C (Figure 2B), indicating that most of the DNA ejected by heating at temperatures lower than 60°C was tethered to the capsids. Several previous studies also observed the phenomenon of some released genomes being attached to the capsid.^10^^,^^11^^,^^13^ After Benzonase treatment, FP_LD_ and FP_HD_ showed similar increases in the proportion of EPs. Interestingly, FP_VP3only_ had a greater EP proportion than FP_LD_ and FP_HD_ after incubation at 45°C–55°C, whereas when incubated at 65°C, FP_LD_ and FP_HD_ had a significantly greater proportion of EPs than FP_VP3only_ (Figure 2C). These results indicated that the ratio of VP1 or VP2 to total VPs barely alters genome release, whereas the VP1u and VP1/VP2 common regions prevent genome release at temperatures below 60°C and facilitate genome release at temperatures greater than 60°C.

**Table 1:** The thermodynamic parameters obtained with nano-DSF for rAAV8 FP_LD_, FP_HD_, FP_VP3only_, and EP

	First transition				Second transition			
	*T*_onset1_ (°C)	*T*_m1_ (°C)	Δ*H*_1_ (10^5^*cal/mol*)	Δ*S*_1_ (10^2^*cal/[K·mol]*)	*T*_onset2_ (°C)	*T*_m2_ (°C)	Δ*H*_2_ (10^5^*cal/mol*)	Δ*S*_2_ (10^2^*cal/[K·mol]*)
FP_LD_	54.6 ± 2.86	62.0 ± 1.01	1.46 ± 0.21	4.34 ± 0.61	71.5 ± 0.15	73.7 ± 0.12	3.21 ± 0.05	9.25 ± 0.15
FP_HD_	53.6 ± 2.04	61.9 ± 0.17	1.43 ± 0.04	4.27 ± 0.12	71.5 ± 0.26	73.6 ± 0.14	3.33 ± 0.41	9.59 ± 1.19
FP_VP3only_	–	–	–	–	71.6 ± 0.04	73.7 ± 0.03	3.27 ± 0.03	9.44 ± 0.08
EP	–	–	–	–	71.3 ± 0.19	73.8 ± 0.14	2.78 ± 0.06	8.02 ± 0.16

The fraction of unfolded protein, $f_{U}$, in two-state unfolding is defined as $K=\frac{f_{U}}{1-f_{U}}$, where K is the equilibrium constant. Gibbs free energy, ΔG, is related to K by ΔG=ΔH−TΔS=−RTlnK, where ΔH, ΔS, R, and T are the enthalpy change, entropy change, gas constant, and temperature, respectively. $f_{U}$ can be written as: (Equation 1) $$f_{U}=\frac{\exp\left(-\frac{\Delta H}{RT}+\frac{\Delta S}{R}\right)}{1+\exp\left(-\frac{\Delta H}{RT}+\frac{\Delta S}{R}\right)}$$

F_350_/F_330_ versus temperature obtained by the nano-DSF measurements can be converted into $f_{U}$ versus the temperature profile. This conversion is achieved by relating experimental values of F_350_/F_330_ signals, E*,* at each temperature for the baselines of the folded ($E_{F}=aT+b$) and unfolded ($E_{U}=cT+d$) states: (Equation 2) $$E=E_{F}+\left(E_{U}-E_{F}\right)f_{U}$$

where a and c are the slope of the baseline for the folded and unfolded states, respectively, and b and d are the intercepts of the baseline for the folded and unfolded states, respectively. Combining Equations 1 and 2, E can be written as Equation 3: (Equation 3) $E=E_{F}+\left(E_{U}+E_{F}\right)\frac{\exp\left(-\frac{\Delta H}{RT}+\frac{\Delta S}{R}\right)}{1+\exp\left(-\frac{\Delta H}{RT}+\frac{\Delta S}{R}\right)}$

Nonlinear least-squares fitting was performed using Equation 3 to determine all parameters. For the first transition, a was set equal to c. For the second transition in FP_LD_ and FP_HD_, a and b were set to the values of c and d obtained from the first transition. The temperatures for $f_{U}$ = 0.5 and 0.05 were defined as *T*_m_ and *T*_onset_, respectively.

### Heat-induced structural changes of low-density, high-density, and VP3-only rAAV8 particles

**Figure 3:**
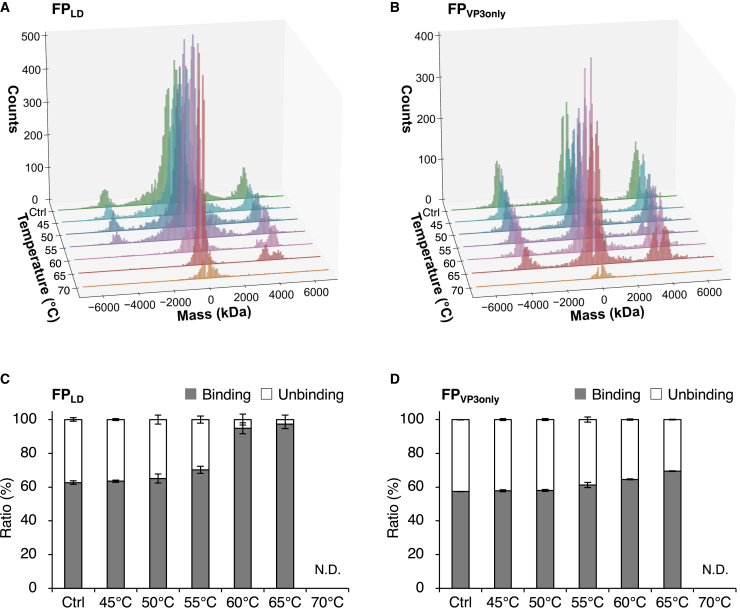
Binding and unbinding properties of rAAV8 FP_LD_ and FP_VP3only_ upon heating (A) MP histograms (expanded for the unbinding side in Figure 2A) for FP_LD_ and (B) FP_VP3only_ after incubation on ice (control), and at 45°C, 50°C, 55°C, 60°C, 65°C, and 70°C. (C and D) The ratio of unbinding and binding particles to the total particle count for (C) FP_LD_ and (D) FP_VP3only_ after incubation on ice (control), and at 45°C, 50°C, 55°C, 60°C, 65°C, and 70°C. Each error bar represents the SD of triplicate measurements.

MP usually observes the binding events of single molecules to a glass surface as dark spots and uses their contrast for mass analysis, but this technique can also detect unbinding events as bright spots.^18^ As shown in Figures 3A and 3B, which depict the extended mass histograms of Figure 2A for the FP_LD_ and FP_VP3only_ samples, respectively, upon heating, binding events are displayed on the positive mass values, whereas unbinding events are displayed on the negative mass values. As described in Figure 2, both FP_LD_ and FP_VP3only_ control samples showed one peak in the range of 2–7 MDa corresponding to FPs, whereas upon heating, above 55°C, two peaks, corresponding to EPs and FPs, respectively, were observed. Binding and unbinding events were repeatedly observed for rAAV8 particles that did or did not contain VP1 and VP2 before heating, with ratios of unbinding particles to total particles of 37.3% and 42.6%, respectively (Figures 3C and 3D). Interestingly, however, when heated, the signal corresponding to unbinding particles diminished to less than 5.2% of the total signal at temperatures greater than 60°C for the FP_LD_ containing VP1/VP2, whereas FP_VP3only_ showed little change compared with the control sample. The behavior of FP_HD_ was similar to that of FP_LD_ (Figure S2), indicating that the difference in the change of binding properties to the glass surface upon heating can be attributed to VP1 or VP2.

**Figure 4:**
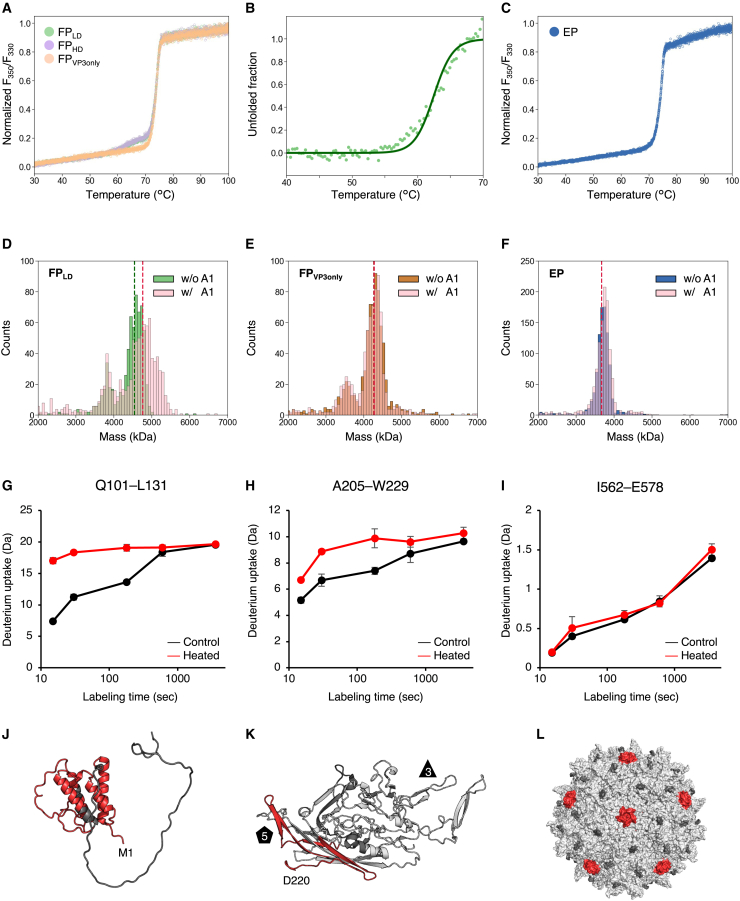
Thermal stabilities of rAAV8 FP_LD_, FP_HD_, FP_VP3only_, and EP; the binding properties of anti-VP1 antibody to FP_LD_, FP_VP3only_, and EP; and the thermal-induced structural changes in rAAV8 (A) Nano-DSF thermograms showing the F_350_/F_330_ of FP_LD_, FP_HD_, and FP_VP3only_. (B) The fitting result of the first transition in the FP_LD_. (C) Nano-DSF thermogram of EP. (D–F) MP histograms of (D) FP_LD_, (E) FP_VP3only_, and (F) EP with and without incubation with anti-VP1 (A1) antibody after heating at 60°C. (G–I) The changes in deuterium uptake by the representative peptides of (G) Q101–L131 in the VP1u region, (H) acetylated A205–W229 in the 5-fold axis, and (I) I562–E578. Each error bar represents the SD of triplicate measurements. (J–L) The differences in deuterium uptake between the control and heated rAAV8s were mapped onto (J) the three-dimensional structure of the VP1 N-termini, (K) the shared VP3 region, and (L) capsids. Compared with control rAAV8s, the red regions show a significant increase in deuterium uptake (>3.281 Da) in heated rAAV8s. Regions where peptides were not detected are shown in gray.

Nano-DSF was then conducted for particles with various VP stoichiometric ratios to capture the unique responses of the particles containing VP1/VP2 to heating (Figure 4A). All of the particles showed a gradual increase in the ratio of the fluorescence intensity at 350 nm to the fluorescence intensity at 330 nm (F_350_/F_330_) from the starting temperature and exhibited large transitions at 70°C–80°C. Importantly, small transitions just before the large transition were observed for both FP_LD_ and FP_HD_, but not for FP_VP3only_. Nonlinear least-squares fitting of the small transition, namely the first transition, in FP_LD_ using a two-state thermal unfolding model revealed that the *T*_onset1_, *T*_m1_, Δ*H*_1_, and Δ*S*_1_ values were 54.6 ± 2.86°C, 62.0 ± 1.01°C, 1.46 ± 0.21 × 10^5^ cal/mol, and 4.34 ± 0.61 × 10^2^ cal/(K·mol), respectively (Figures 4B and S3; Table 1). The thermodynamic parameters of globular proteins at 62°C, reported in a previous pioneering study of the calorimetry of proteins by Privalov and Khechinashvili, were approximately 5.7–8.7 cal/g for Δ*H* and 1.7–2.7 × 10^˗2^ cal/(K·g) for Δ*S*.^19^ Thus, we transformed the thermodynamic parameters obtained in the first transition for FP_LD_ to the same units as reported previously,^19^ using the mass of only the VP1u, the VP1u and VP1/VP2 common regions (VP1 N-termini), or the VP1/VP2 common region only. When the mass of the VP1 N-termini (i.e., 22 kDa) was used, we obtained values of 6.7 ± 1.0 cal/g for Δ*H*_1_ and 2.0 ± 0.3 ×10^˗2^ cal/(K·g) for Δ*S*_1_, which were in the range of the thermodynamic parameters for globular proteins. This finding was consistent with an earlier cryoelectron microscopy observation.^20^ The thermodynamic parameters for the FP_HD_ were almost identical to those for the FP_LD_ (Table 1). These results indicated that the VP1 N-termini were folded in the presence of encapsidated DNA below *T*_onset1_ and that the first transition corresponds to the unfolding of the VP1 N-termini, which was further supported in this study, as described later.

VP1u has been reported to be exposed outward from the inside of the capsid upon heating, while the capsid maintained the assembled state, as revealed by dot blotting analysis using anti-VP1 (A1) which specifically recognizes the VP1u region, and anti-AAV capsid antibodies.^9^^,^^20^^,^^21^^,^^22^ To confirm the relationship between the unfolding and externalization, we examined whether A1 antibodies were bound to rAAV8 capsids before and after heating at 60°C, a temperature at which the VP1 N-termini begin to unfold, using FP_LD_ and FP_VP3only_. We found no difference in the mass with or without A1 for all particles before heating (Figure S4). By contrast, an increase in mass due to the binding of A1 to the particle was observed only for FP_LD_ after heating (Figures 4D and 4E), indicating that VP1u was exposed outward simultaneously with or following the unfolding process of the VP1 N-termini, whereas the particles retained the assembled capsid state.

Interestingly, despite the incorporation of both VP1 and VP2 into the EP capsid, neither the first transition in the nano-DSF measurement (Figure 4C) nor the interaction with A1 antibody in the MP measurement (Figure 4F) was observed for EPs, which possessed no encapsidated genome. The absence of the first transition indicated that the VP1 N-termini had an intrinsically unfolded structure in EPs, which has been supported by recent HDX-MS studies that revealed the higher deuterium uptake of VP1u in EPs than FPs.^23^^,^^24^ Thus, VP1u externalization requires a push-out mechanism by the encapsidated genome, which is consistent with the findings of a previous study.^20^ The *T*_onset_ and *T*_m_ of the major transition, namely the second transition, were almost the same among all of the samples with *T*_onset2_ at 71°C–72°C and *T*_m2_ at 74°C, indicating that the second transition corresponded to the unfolding of the shared VP3 region and was not affected by the DNA packaging state (Figure S3; Table 1).

HDX-MS was subsequently conducted to examine the structural dynamics of rAAV8 at the peptide level upon heating. The uptake of deuterium by rAAV8 heated at 60°C, which is close to the *T*_m1_ and below the *T*_onset2_, was compared with that of unheated samples (control). We found that the samples heated at 60°C showed an increase of deuterium uptake in the VP1u region, for example peptide Q101–L131 (Figures 4G and 4J), which indicates the unfolding of the VP1 N-termini upon heating at *T*_m1_, as expected. Interestingly, most of the shared VP3 regions did not show a change in deuterium uptake, such as I562–E578, whereas a 5-fold axis region, such as acetylated A205–W229, showed a significant increase in deuterium uptake (Figures 4H, 4I, 4K, and 4L). Because the shared VP3 region was folded and the capsid was retained at temperatures lower than the *T*_onset2_, the increase in deuterium uptake in the 5-fold axis region may arise from increased structural dynamics. The narrow pore at the 5-fold axis in rAAV is the channel used to externalize the VP1 N-terminus.^20^^,^^21^^,^^25^ Therefore, the increase in structural dynamics was due to the expansion of the pore at the 5-fold axis for the externalization of the VP1u region, as shown in Figure 4D.

### Particle distribution upon heating in the entire rAAV8 solution

**Figure 5:**
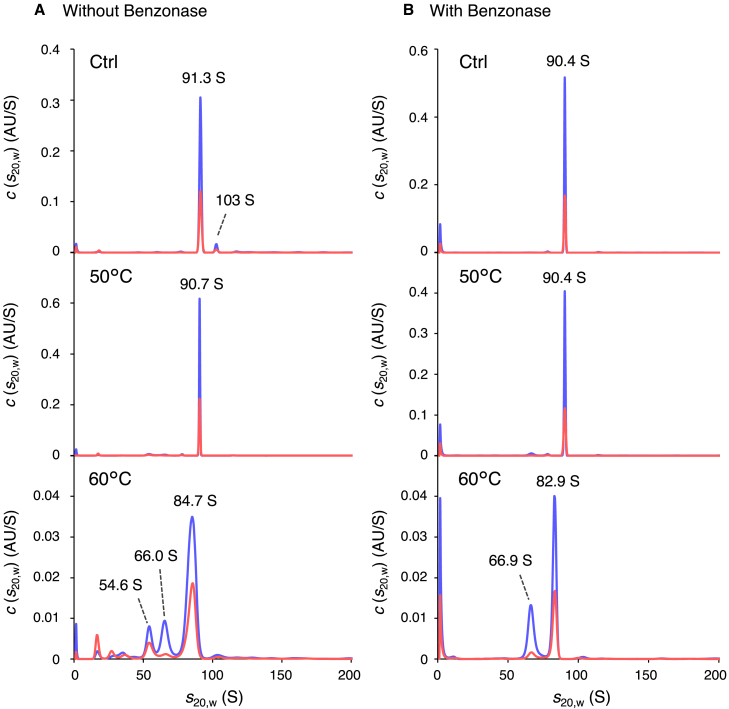
Particle distributions in the rAAV8 sample solutions upon heating Sedimentation profiles of (A) Benzonase-untreated and (B) Benzonase-treated rAAV8 solutions after incubation on ice (control) (top), 50°C (middle), and 60°C (bottom). The blue and red lines represent the profiles obtained at an absorbance of 230 and 260 nm, respectively.

Although MP is a powerful technique for analyzing particle distribution in samples of limited volume, assessing large aggregates that exceed the measurable mass range, and distinguishing molecules with different shapes, but the same mass is challenging. Therefore, in the present study, rAAV8 solutions were heated and then analyzed by SV-AUC with multiwavelength detection to allow for particle characterization even in the presence of aggregates based on spectral information. Because capsids and ssDNA absorb UV light differently at 230 nm and 260 nm in UV detection,^26^ the ratio of the peak area obtained by detection at 260 nm to the peak area obtained by detection at 230 nm (A_260_/A_230_) can be used to identify the components of each peak. It is worth noting that the rAAV8 solutions were not fractionated separately by the VP1/VP2 ratio because the MP and nano-DSF results in the present study revealed that the ratio of VP1 and VP2 to the whole VPs may hardly change the genomic release behavior and thermodynamic parameters. Samples treated by incubation at 50°C and 60°C were centrifuged at 3,000 rpm with an Optima AUC analytical ultracentrifuge, resulting in decreased absorption at 230 nm to 6.2% at 50°C and 27.2% at 60°C compared with the control sample (Figure S5). These results indicated that some of the rAAV8 particles formed large aggregates upon heating, as found in a previous CD-MS study^13^; such aggregates sedimented at 3,000 rpm; and the sedimentation profiles obtained with SV-AUC were related to the rAAV8 monomeric particles or small aggregates that sedimented at 20,000 rpm. As shown in Figure 5A, the peak for FPs was observed at 91.3 S in the sedimentation profiles for the control sample; the A_260_/A_230_ value of the peak for FPs in this sample was 0.47. The minor peak at 103 S, with an A_260_/A_230_ of 0.52, disappeared after Benzonase treatment (Figure 5B), suggesting that this peak corresponded to FPs with DNA fragments. Incubation at 50°C did not significantly change the particle distribution, and after Benzonase treatment, there was no change in sedimentation profile except for an increase near the meniscus that may have originated from Benzonase (Figures 5A and 5B). By contrast, incubation at 60°C produced two major additional peaks at 54.6 S and 66.0 S along with minor peaks at 15–50 S, besides the shift of the original major peak from 91.3 S to 84.7 S, and after Benzonase treatment, the minor peaks at 15–50 S and the peak at 54.6 S disappeared (Figure 5B).

The A_260_/A_230_ value of the peak at 84.7 S in the 60°C-heated samples was 0.47, which was the same as that of the peak at 91.3 S in the control sample. Because the A_260_/A_230_ value indicates that the peak components are a combination of proteins and the genome, if the VP multimers are formed at an 84.7 S peak at disassembly, this peak should have disappeared after Benzonase treatment; however, the peak at 82.9 S with an A_260_/A_230_ value of 0.47 was observed after Benzonase treatment, indicating that the components with a peak at 84.7 S should correspond to the FPs. Thus, the decrease in *s* values may correspond to the ejection of a portion of the genome and/or a slight loss of VPs that caused a negligible change in the A_260_/A_230_ value. Notably, Orbitrap CD-MS analysis showed a mass shift of the FP peak from 4.55 ± 0.01 MDa in the control sample to 4.45 ± 0.01 MDa in the 60°C-heated samples (Figure S6). The 60°C-heated samples were treated with Benzonase, fractionated to EPs and FPs by CsCl DG-UC, and the fraction of FPs was analyzed by CE-LIF (Figure S7). One major peak corresponding to the designed genome was observed for both the control and 60°C-heated FPs, with no shift of the major peak. It is worth noting that no peak corresponding to the ssDNA fragments, which has been reported to cause a decrease in mass at higher temperatures,^27^ was observed in the samples used in the present study. The discrepancy in the samples may arise from the different cell lines used for rAAV production (Sf9 and HEK293 cells). The results of CE-LIF and CD-MS indicate that the shift in the *s* value was caused not by the ejection of a portion of the genome but by a slight loss of VPs. However, based on the Svedberg equation, $s=M\left(1-\bar{v}\rho \right)/N_{A}f$, where s, M, $\bar{v}$, ρ, $N_{A}$, and f are the sedimentation coefficient, buoyant molecular weight, partial specific volume, buffer density, Avogadro constant, and frictional coefficient, respectively, and the mass shift was 0.1 MDa, the expected *s* value in the 60°C-heated samples was 89.3 S. Therefore, the shift from 91.3 to 84.7 S suggested that the structural changes in capsids that altered the frictional coefficient occurred in addition to VP loss. Considering that the A_260_/A_230_ value and *s* value for the peak of purified EPs were 0.10 and 65.6 S (Figure S8), respectively, the peak at 66.0 S with an A_260_/A_230_ value of 0.16 for the sample after heating could be assigned as EPs or EPs with tiny DNA fragments. By contrast, the peak at 54.6 S with an A_260_/A_230_ of 0.58 for the sample after heating was considered to represent particles that had ejected the entire length of ssDNA encapsidated before heating, which was further supported by the effect of Benzonase treatment of the 60°C-heated sample, resulting in the disappearance of the peak at 54.6 S and an increase in the intensity of the EP peak at 66.9 S (Figure 5B).

### Heat-induced genome release of full and overpackaged rAAV8s

**Figure 6:**
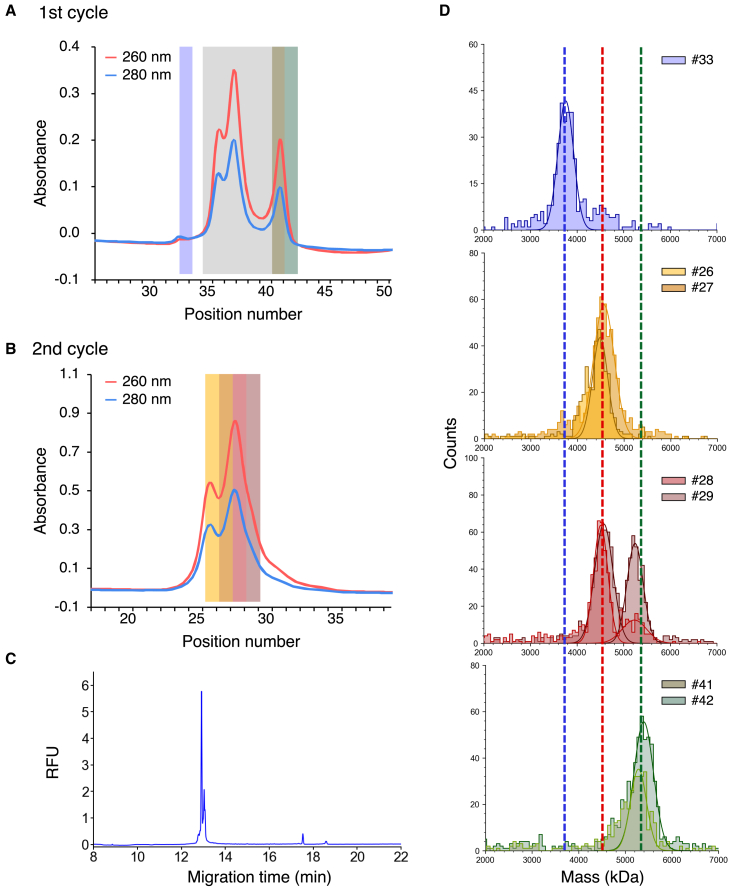
Characterization of the commercial rAAV8 sample (A and B) CsCl DG-UC equilibration profiles of the commercial rAAV8 sample after the (A) first cycle and (B) the second cycle. The red and blue lines represent the profiles obtained at an absorbance of 260 and 280 nm, respectively. (C) CE-LIF electropherogram of ssDNA encapsidated in OPs in fractions 41–42 after the first cycle of CsCl DG-UC. (D) MP histograms of fraction 33 after the first cycle (top), fractions 26–27 after the second cycle (upper middle), fractions 28–29 after the second cycle (lower middle), and fractions 41–42 after the first cycle (bottom).

Finally, genome release was assessed in a fractionated sample of commercially available rAAV8. rAAVs are generally produced as a mixture of EPs and FPs, and frequently contain partial particles that encapsidate the partly designed genome and OPs, which have a larger mass than FPs. A commercial rAAV8 sample showed four peaks after the first cycle of CsCl DG-UC (Figure 6A). Fractions 35–40, which correspond to overlapping major peaks, were then mixed, followed by a second cycle of CsCl DG-UC, resulting in two distinct peaks (Figure 6B). Similarly, the particle size distribution for each fraction determined by MP measurements revealed that the commercial sample contained EPs with a theoretical mass of 3.73 MDa, FPs with a theoretical mass of 4.51 MDa encapsidating the full-length genome of 2,521 bases, and OPs (Figure 6D). For the first cycle, fraction 33 contained EPs, and fractions 41 and 42 contained OPs. Unexpectedly, the ssDNA distribution encapsidated in OPs in the mixture of fractions 41 and 42, as analyzed by CE-LIF, showed a major peak at 12.9–13.0 min, corresponding to DNA fragments with 581–607 bases (Figure 6C). The small peaks at 17.5 min and 18.6 min, corresponding to the full-length designed genome and a larger genome than the designed genome, respectively, were also observed. The theoretical masses of EPs and FPs are 3.7 MDa and 4.5 MDa, respectively, when the capsid comprises VP1:VP2:VP3 = 5:5:50 and encapsidates the full-length genome with 2,521 bases and 0.8 MDa. Because the mass of OPs was determined as 5.3 MDa by MP, OPs possibly encapsidate one large genome of approximately 4.8 kb and 1.6 MDa, two full-length genomes, or a combination of full-length genome and four DNA fragments of approximately 590 bases and 0.2 MDa. However, because two peaks corresponding to the full-length genome and the larger genome, respectively, produced substantially smaller intensity peaks than the peak of DNA fragments, most OPs were the particles that packed with DNA fragments up to the encapsidation size limit. After a second cycle of CsCl DG-UC, fractions 26 and 27 contained FPs, and fractions 28 and 29 contained both FPs and OPs. OPs in fractions 28 and 29 in the second cycle were considered to encapsidate the same length of ssDNA as OPs in fractions 41 and 42 from the first cycle but had different VP stoichiometric ratios, as described for FPs in Figure 1. Thus, fractions 28 and 29 from the second cycle of CsCl DG-UC were collected and used in the following experiment as a model sample that contains both FPs and OPs.

**Figure 7:**
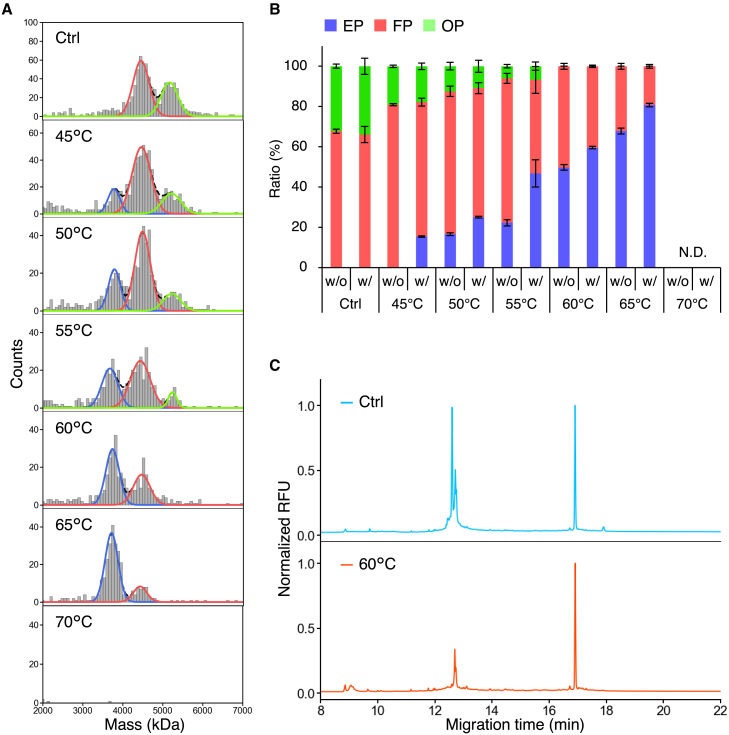
Observation of heat-induced genome release from the fractionated rAAV8 sample (A) MP histograms of Benzonase-treated rAAV8 samples after incubation on ice (control), and at 45°C, 50°C, 55°C, 60°C, 65°C, and 70°C. The blue, red, and green lines represent the areas of EPs, FPs, and OPs, respectively. (B) The ratio of each particle to total particles in the rAAV8 sample with or without Benzonase treatment after incubation on ice (control), and at 45°C, 50°C, 55°C, 60°C, 65°C, and 70°C. Each error bar represents the SD of triplicate measurements. (C) CE-LIF electropherograms of ssDNA encapsidated in FPs and OPs before (top) and after (bottom) incubation at 60°C.

The collected sample was incubated on ice (control), and at 45°C, 50°C, 55°C, 60°C, 65°C, and 70°C for 15 min, and the particle size distributions were assessed by MP (Figures 7A and 7B). The proportion of EPs increased with increasing incubation temperature, whereas the proportions of FPs and OPs decreased, with no OPs detected at 60°C. After incubation at 70°C, no particles were detected in the range of 2–7 MDa. Because the ratio of VP1 or VP2 to total VPs barely altered genome release based on the results from FP_LD_ and FP_HD_, the FPs in the collected sample should show genome release consistent with that of FP_LD_ and FP_HD_. Thus, it was considered that OPs released their genome at 45°C–50°C and were then observed as EPs. Benzonase treatment after the incubation increased the proportion of EPs, indicating that some ejected ssDNA was tethered to the particles, which had similar molecular masses to those of FPs and OPs (Figure 7B). The size distributions of the encapsidated ssDNA after Benzonase treatment were measured by CE-LIF. The result showed a broad peak at 12.6–12.7 min and a sharp peak at 16.9 min (Figure 7C) for the control sample. The peak at 12.6–12.7 min was attributed to ssDNA fragments with 576–607 bases considered encapsidated in OPs on the basis of the fractionation and characterization results (Figure 6C). The peak at 16.9 min was attributed to ssDNA with 2,285 ± 6 bases, corresponding to the full-length designed genome with 2,521 bases, which was encapsidated in the FPs. After incubation at 60°C, as normalized by the peak area at 16.9 min, the DNA fragment peak area was 57% less than that of the control, indicating that the DNA fragments were preferentially released by heating, which was consistent with the lack of detection of OPs in the sample after incubation at 60°C (Figure 7C). The remaining peak was considered to contain ssDNA fragments encapsidated in FPs with a full-length ssDNA genome or in OPs at a level that could not be resolved by MP or was less than its limit of detection. Interestingly, we observed no peak corresponding to DNA fragments ranging between 579 and 609 bases and full-length ssDNA, which was expected to be generated from particles that had stopped ejecting ssDNA partway through (Figure 7C). These findings indicated that under the heating conditions in the present study, no particles stopped ejecting ssDNA partway through, and the particles existed in three states: capsids that did not eject ssDNA, capsids that completely ejected ssDNA and had the genome tethered to their surface, and capsids that fully released their encapsidated genome and were observed as EPs.

## Discussion

The release of rAAV genomes has been studied; however, previous findings have been inconsistent. Here, we focused on the influence of various VP stoichiometric ratios in capsids on the heat-induced genome release of rAAV. Regarding the reported discrepancy as to whether rAAV releases the capsid genome while retaining the capsid,^9^^,^^10^^,^^11^ we conclude that by using highly purified rAAV8 FPs that did not contain EPs, some particles completely released their encapsidated genome upon heating and were observed as EPs. Moreover, the characterization of the samples, which included both FPs and OPs, revealed that OPs were predominant, which encapsidate multiple short lengths of ssDNA up to the packaging capacity of the rAAVs. The encapsidated short DNA fragments were released below the *T*_onset1_ and behaved differently from the full-length genome. These findings suggest that differences in the encapsidated genome sizes and VP1 N-terminal thermal stabilities can be attributed to the discrepancy in genome length-dependent genome release observed in previous studies.^9^^,^^12^^,^^13^

The present study clarified that the extent of genome release was not significantly affected by the difference in the stoichiometric ratio of VP1/VP2 but changed depending on whether VP1/VP2 was incorporated into the capsids. Nano-DSF and MP using A1 antibody revealed that the majority of VP1 N-termini were folded and located within the capsid below the *T*_onset1_, and MP analysis revealed that a greater percentage of the genome was ejected by FP_VP3only_ than by FP_LD_ and FP_HD_ (FP_VP1/VP2/VP3_). These findings indicated that folding of the VP1 N-termini could prevent the genome from entering the pore at the 5-fold axis. By contrast, heating at a temperature close to the *T*_m1_ led to the unfolding of the VP1 N-termini and to the expansion of the pore at the 5-fold axis for the externalization of unfolded and linearized VP1 N-termini, as shown by HDX-MS and MP using A1 antibody. A greater level of genome ejection at 65°C by FP_VP1/VP2/VP3_ than FP_VP3only_ could be explained by an expanded pore at the 5-fold axis in FP_VP1/VP2/VP3_ compared with FP_VP3only_, indicating that unfolding of the VP1 N-termini facilitates genome ejection. The short ssDNAs in OPs were found to be released preferentially to the designed-length genome in FPs in MP measurements, which could be explained by the higher mobility of short-length DNA compared with the designed-length genome. The diffusion coefficient of ssDNA in an aqueous solution is smaller for longer ssDNAs in the presence of a denaturant.^28^ Therefore, ssDNAs of approximately one-quarter of the size of the full-length genome in OPs, as confirmed by CE-LIF, would have greater mobility than the full-length genome in FPs to reach the pore and would therefore be more easily ejected from the capsid.

The MP measurements of FP_VP1/VP2/VP3_ and FP_VP3only_ revealed that heating above 60°C resulted in fewer particles being detected at the unbinding side for FP_VP1/VP2/VP3_ only. The unfolding of the VP1 N-termini and the binding of A1 antibodies to the accessible linearized epitope located at the VP1u in rAAV8 particles were observed by nano-DSF and MP, respectively, at this temperature. Protein adsorption to glass surfaces is usually governed by the pH and ionic strength of the solvent, as well as the nature of the protein itself. rAAV8 is considered to weakly interact with the glass surface under the conditions for MP measurement because the entire particle is megadalton-sized and the capsid surface formed by the shared VP3 region is devoid of charge bias with scattered positive and negative surfaces at pH 7 with 150 mM NaCl (Figure S9). However, the VP1 N-terminus, which has a mass of 22 kDa, has positively biased regions, especially in part of the VP1/VP2 common region, resulting in strong binding to the negatively charged glass surface if the region is exposed. Therefore, the reduction in unbinding rAAV8 particles from the glass surface above the *T*_onset_ for VP1 N-termini unfolding may cause the externalization of the VP1/VP2 common region.

The loss of a number of particles was detected in the 0–2,000 and −2,000–0 kDa ranges as the number of counts for 1 min of unbinding particles, which decreased only for FP_VP1/VP2/VP3_. We consider that before heating, most particles detected in these regions do not reflect the components contained in the samples and are instead an artifact of the MP analysis because signals in this region were observed with MP even for samples where no peak was observed between 0 and 90 S in the SV-AUC analysis (Figure S10A).

The midpoint temperature at which the VP3 shared region unfolds (*T*_m_) and leads to capsid disassembly is approximately 74°C on the basis of the nano-DSF observations, regardless of the encapsidation state of the genome or the VP stoichiometric ratio. However, the capsid was not observed by MP at 70°C for any of the samples, which could be due to the formation of aggregates before or immediately after capsid disassembly. SV-AUC revealed that rAAV8 formed large aggregates after incubation at 60°C, based on the decrease in A_230_ of 27.2% at 3,000 rpm rotation, whereas MP gave inconsistent results regarding the detection of aggregates (Figure S10B). It is thought that if the aggregates adsorbed onto the glass surface, they could be detected even if their mass was outside the measurable range. Notably, a CD-MS study showed a mass distribution of aggregates above 5–100 MDa.^13^ However, the aggregates may not have been detected during the 1-min measurement, which was too short to allow for slow diffusion in the solution to the detector surface. Each particle concentration in the 60°C-heated samples was calculated using the extinction coefficient of EPs for the EP peak and the extinction coefficient of FPs for the two peaks assigned to EPs with the ejected ssDNA and FPs (Equation S1). The total particle concentrations of EPs, EPs with the entire length of ssDNA ejected, and FPs in the 60°C-treated samples were reduced to 29.5% that of the FPs in the control, which was only a 2% difference from the decrease in absorbance at 3,000 rpm. This result indicated that the decrease in FPs from the control may be primarily due to the formation of aggregates, and that most remaining FPs exist in monomeric particles as FPs with an altered structure or EPs that have ejected or released the genome, with only 5% of particles being completely disassembled. Additionally, no peak was observed in the 100–200 S range after SV-AUC of the samples heated at 50°C and 60°C, indicating that rAAV8 particles do not form small aggregates but rather large aggregates immediately upon heating.

The MP and SV-AUC analyses revealed that rAAV8 particles ejected their encapsidated genomes upon heating while retaining an assembled capsid, and that some ejected genomes were tethered to the surface of their capsids, whereas other capsids were completely separated from their ejected genomes and observed as EPs. Moreover, SV-AUC successfully distinguished particles with two different molecular shapes that were detected as FPs based on their masses, as determined by MP. The frictional ratio, *f/f_0_*, for the main particles (FPs) of 91.3 S was experimentally determined to be 1.21 in the control sample. On the basis of the Svedberg equation and the small mass shift observed by CD-MS, *f/f_0_* was calculated to be 2.07 for the particles of 54.6 S and 1.33 for those of 84.7 S, in the sample after heating at 60°C. Hydrated spherical proteins have an *f/f_0_* of approximately 1.2,^29^ indicating that the rAAV8 particles have a relatively globular shape before heating, whereas after heating present two structures an elongated ellipsoid shape corresponding to particles tethering the ejected ssDNA and an almost globular shape that differs from that of the control sample. The appearance of the particles at 54.7S is consistent with a previous study that found an increase in the hydrodynamic radius or the radius of gyration of heavy capsids, which was generated when FPs were heated,^30^ and a CD-MS study that showed highly charged ions were observed for heated rAAV.^13^

The present study clarified the relationship between the thermal unfolding of VP1 N-termini and the heat-induced genome release of rAAV. Below *T*_onset2_, the thermal unfolding of VP1 N-termini was closely monitored, which is related to genome release upon heating. We found that folded VP1 N-termini prevented the release of the full-length genome, whereas the unfolding of the VP1 N-termini facilitated genome release. Above the *T*_onset1_ and below the *T*_onset2_, rAAV8 particles ejected their genome from almost intact capsids, partly as monomeric particles and partly as aggregates, and the monomeric particles existed in three states: capsids that do not eject ssDNA, capsids that fully release their encapsidated genome and are observed as EPs, and capsids that completely eject the genome tethering to their surface and form an elongated ellipsoid shape. rAAV8 particles ejected DNA that was much shorter than the full-length genome with little or no conformational change in the capsid after heating below the *T*_onset1_, whereas above the *T*_m1_, rAAV8 particles ejected their genome with externalization of the VP1 N-termini in advance of capsid disassembly. These findings provide new insights into virus genome release with a focus on VPs of rAAV at the molecular level and present a new approach to rAAV development from the perspective of VP1 N-terminal stability.

A study focusing on pH-dependent genome ejection by rAAV2 and rAAV5 was recently published.^31^ The paper reported that upon heating to below 55°C, AAV5-VP3 showed a lower viral titer than AAV5 wild-type, whereas upon heating above 60°C, AAV5-VP3 showed a higher viral titer than AAV5 wild-type. This was consistent with our findings for rAAV8 that below the *T*_m1_ of approximately 60°C, FP_VP3only_ showed higher genome ejection compared with FP_VP1/VP2/VP3_, whereas above the *T*_m1_, FP_VP3only_ showed lower genome ejection compared with FP_VP1/VP2/VP3_. Moreover, the paper reported that heating impacted the peripheral density surrounding the core rod at the 5-fold axis, which was consistent with the changes in deuterium uptake at the 5-fold axis upon heating observed by HDX-MS in our study. The findings of our study, combined with these recently published data, provide a deeper understanding of the mechanism of genome ejection by rAAV.

## Materials and methods

### rAAV8 preparation

rAAV8 vectors were purchased from VectorBuilder (Chicago, IL, USA). rAAV8 vectors were generated in-house using a triple plasmid. In brief, Rep2 and Cap8, or pAAV-Rep2 and Cap8_VP3_only_ (the plasmid with the VP1 and VP2 start codons mutated as reported previously^32^), pAd helper, and transgene (CMV-EGFP) plasmids (VectorBuilder, vector ID: VB010000-9394npt) were cotransfected at a ratio of 1:1:1 into suspended HEK293F cells (Viral Production Cells 2.0, Thermo Fisher Scientific, Waltham, MA, USA). rAAV8 vectors from the cultured transfected cells and the medium were harvested 96 h post-transfection and purified by affinity chromatography using AAVX columns (Thermo Fisher Scientific). One cycle of CsCl DG-UC was then used to separate FPs and EPs.

### Two cycles of CsCl DG-UC purification

Two cycles of CsCl DG-UC were performed to remove the few remaining EPs mixed with FPs. The purified in-house and commercial rAAV samples were transferred to the first cycle of CsCl DG-UC. The virus band containing the enriched FPs was extracted and subjected to another round of ultracentrifugation. Finally, the samples were dialyzed with 1× phosphate-buffered saline (PBS) supplemented with 200 mM NaCl and 0.001% poloxamer 188 at pH 7.4. Poloxamer 188 (Kolliphor P188 Bio; P188) was a gift from BASF (Ludwigshafen, Germany).

### Capillary gel electrophoresis using sodium dodecyl sulfate with UV detection

rAAV8 samples for CE-SDS analysis were prepared following a previously reported procedure.^33^ rAAV8 solutions in 10 μL (5.0 × 10^10^ viral genome [vg]) were denatured and buffer-exchanged using the following protocol. The final sample collected was diluted with 50 μL of Milli-Q water for injection. A PA 800 Plus system (Sciex, Framingham, MA, USA) was used for the CE analyses. The prepared samples were injected by water plug sample stacking. To determine the VP stoichiometric ratio, the areas of the peaks reflecting the UV absorbance of peptide bonds and amino acids under denaturing SDS conditions, as detected at 214 nm using a photodiode array detector, were divided by the molar extinction efficient of VPs at 214 nm.

### Capillary gel electrophoresis with laser-induced fluorescence detection

A solution of rAAV8 in 10 μL (1 × 10^11^ vg for untreated and 4 × 10^11^ vg for heat-treated samples), 3 μL of 10 × DNase buffer (AM8170G; Thermo Fisher Scientific), 1.5 μL of Benzonase (catalog no. E1014; Sigma-Aldrich, St. Louis, MO, USA), and 15.5 μL of 1× PBS with 0.001% poloxamer 188 were mixed to obtain a final volume of 30 μL. Then, the sample was incubated at 37°C for 30 min. Subsequently, 30 μL of DNase-treated rAAV8 was mixed with 10 μL of 500 mM EDTA (Nippon Gene, Toyama, Japan), 55 μL of 1× PBS with 0.001% poloxamer 188, and 5 μL of 20 mg/mL Proteinase K (catalog no. 19131, QIAGEN, Hilden, Germany) to obtain a final volume of 100 μL. Next, the mixture was incubated at 55°C for 60 min and then heated at 95°C for 20 min, followed by centrifugation to collect the lysate. The ssDNA was subsequently purified according to a protocol for the QIAquick PCR Purification Kit (QIAGEN) and used as the final collected sample. A PA 800 Plus system (Sciex) was used for CE analyses. The prepared samples were loaded via electrokinetic injection, and the fluorescence induced by 488-nm laser excitation of the electrophoretically separated sample components was detected by a photometer using a 520-nm emission filter.

### Orbitrap-based charge detection mass spectrometry

Each sample (20 μL) was applied to a Micro Bio-Spin6 Column (Bio-Rad, Hercules, CA, USA) and exchanged into 200-mM ammonium acetate buffer adjusted to pH 7.4. Samples (5 μL) were loaded into glass capillaries and coated with gold in-house for nanoelectrospray ionization. Mass analysis was conducted using the Direct Mass Technology mode on a Q Exactive UHMR Hybrid Quadrupole-Orbitrap MS System (Thermo Fisher Scientific) with an ion transfer target and detector optimization of high m/z, a spray voltage of 1.2 kV, a capillary temperature of 350°C, and a desolvation voltage of −15 V. The trapping gas pressure was in the range of 2.50–2.77 × 10^˗10^ or 4.50–4.62 × 10^˗10^ mbar using SF6 gas. Data were acquired in triplicate at 50 K at m/z 400 resolution with an m/z range of 20,000–40,000 for 20 min or 10,000–40,000 for 30 min, processed by STORIBoard software version 1.0 (Proteinaceous, Chicago, IL), and analyzed using an in-house Python 3 script to obtain mass values.

### Sample preparation for MP

For heat-induced genome release studies, 34 μL samples of an rAAV8 solution (2 × 10^12^ vg/mL) were heated at the desired temperatures for 15 min and quenched on ice. To prepare the samples with DNA digestion, 17 μL samples of treated rAAV8 solutions, 2.0 μL of 10× DNase I buffer, and 1.0 μL of Benzonase were mixed. To prepare samples without DNA digestion, 1.0 μL of Milli-Q water was substituted for Benzonase. The mixtures were subsequently incubated at 37°C for 30 min, and the reaction was quenched on ice. Then, 1.0 μL of 500 mM EDTA with 0.2 w/v% poloxamer 188 was added to the AAV sample.

For VP1u detection with A1 antibody, 12 μL samples of rAAV8 solutions (1.0 × 10^13^ vg/mL) were incubated at room temperature or 60°C for 15 min, and 6 μL of each were dispensed. Subsequently, 24 μL of 50 μg/mL A1 antibody (catalog no. 61056; Progen, Heidelberg, Germany) or 1× PBS as a control were mixed to obtain final rAAV8 and A1 concentrations of 2.0 × 10^12^ vg/mL and 40 μg/mL, respectively. The rAAV8 and A1 mixture was subsequently incubated at room temperature for 1 h at 300 rpm on a ThermoMixer C (Eppendorf, Hamburg, Germany).

### MP measurement and analysis

MP measurements were conducted using a TwoMP instrument (Refeyn, Oxford, UK). Coverslips (24 × 50 mm precision; Thorlabs, Newton, NJ, USA) were prepared by cleaning with Milli-Q water and ethanol. A piece of precut 2 × 3 culture well gasket (Grace Bio-Labs, Bend, OR, USA) was placed onto the coverslip. For MP measurements, 18 or 12 μL of 1× PBS for heat-induced genome release and VP1u detection, respectively, was loaded into the well created by the gasket, and the focus was automatically adjusted. Then, 2 or 8 μL of the sample was added to the same wells, respectively, to produce a final volume of 20 μL, and the samples were mixed by pipetting. Next, 60 s of movie data were recorded using AcquireMP software version 2.5.0 (Refeyn). The MP movie files were analyzed using DiscoverMP software version 2.5.0 (Refeyn). The molecular mass of each sample was estimated from the MP contrast distribution by applying the contrast-to-mass calibration obtained using BSA (catalog no. A2153; Sigma-Aldrich), apoferritin (catalog no. A3660; Sigma-Aldrich), and thyroglobulin (catalog no. T9145; Sigma-Aldrich). For further evaluation, the mass distribution was analyzed using a Python script created in-house. The histograms of the mass distributions were peak-fitted with a Gaussian function for EPs, FPs, and OPs using the in-house Python script. A previous paper has shown that in two MP measurements, noise components appear on the left side of the dominant peak.^17^ The proportion of noise to the sum of FPs and noise was 7.7% ± 2.3% when the peak was fitted to a Gaussian function for the noise in the region where EPs were present in the control FP sample. In the present study, EPs were considered significantly increased when the ratio of EPs was greater than this proportion at +3 standard deviations, or 14.8%.

### Nano-differential scanning fluorimetry

A solution of rAAV8 (4.0 × 10^12^ vg/mL) was formulated in 1× PBS with 200 mM NaCl and 0.001% poloxamer 188. The rAAV8 samples were used to fill standard-grade capillaries for DSF. A Prometheus NT.48 instrument (NanoTemper Technologies, Munich, Germany) was used to determine the thermal stability of the AAV capsid. The system detects the intrinsic fluorescence intensity at 330 and 350 nm after excitation at 280 nm. We performed three consecutive temperature scans of the same sample (3 × 10 μL) from 30°C to 100°C with a linear ramp of 1°C per min. Thermodynamic parameters were determined by nonlinear least-squares fitting of the two-state unfolding model to the obtained nano-DSF data as described in a previous report,^34^ using the in-house Python script.

### Hydrogen/deuterium exchange mass spectrometry

A solution of rAAV8 (2.9 × 10^13^ vg/mL) was incubated at 60°C for 15 min, followed by centrifugation at 10,000 ×g for 10 min to remove large aggregates, and was then used as the heated rAAV8 sample. Both non-heated (control) and heated samples were labeled by a 10-fold dilution with D_2_O buffer. The D_2_O buffer was prepared by dissolving dried 10× PBS and 200 mM NaCl with 10 times the amount of D_2_O (Iwatani, Osaka, Japan) and adjusting to pH_read_ 6.6 (pD 7.0). All labelings were performed at 4°C to slow the HDX rate due to the rapid deuterium uptake of the highly dynamic VP1u region.^23^ The labeling reaction was quenched after 15, 30, 180, 600, and 3600 s by transferring the labeled sample into ice-cold quenching solution at a ratio of 0.6:1. The quenching solution comprised 8 M guanidine hydrochloride (Fujifilm Wako Pure Chemicals), 8 mM Tris(2-carboxyethyl)phosphine hydrochloride (Nacalai Tesque, Kyoto, Japan), and 200 mM NaH_2_PO_4_ (Fujifilm Wako Pure Chemicals), and was adjusted to pH 2.5 with HCl (Fujifilm Wako Pure Chemicals) under the quenching conditions. The quenched samples were subsequently diluted 2.5-fold with ice-cold formic acid in water adjusted to pH 2.5 before injection into an liquid chromatography system (Ultimate 3000, Thermo Fisher Scientific). All the aforementioned procedures were performed using the HDX-PAL system (Trajan, Morrisville, NC, USA), except for the labeling and quenching procedures for the shortest 15-s labeling condition, which were performed manually. Online digestion was performed using the dual protease column (immobilized protease type XIII/pepsin column [w/w, 1:1], 2.1 mm × 30 mm, NovaBioAssays, Woburn, MA, USA) for 4 min in formic acid in water adjusted to pH 2.5. Desalination and separation were conducted using a trap column (PepMap300 C18 5 μm, 1 mm × 15 mm) and an analytical column (Hypersil GOLD, 1 mm × 50 mm, 1.9 μm particle size; Thermo Fisher Scientific), respectively. Solutions of 0.1% formic acid in water (Kanto Chemical, Tokyo, Japan) and 0.1% formic acid in acetonitrile (Kanto Chemical) were used for mobile phases A and B, respectively. The peptic peptides were separated using 16 min gradients. Specifically, %B was rapidly increased from an initial setting of 8%–17% over the first minute, and then gradually increased to 30% over the next 15 min. The eluted peptides were analyzed using a Q Exactive HF-X mass spectrometer (Thermo Fisher Scientific) with an electrospray ionization device operated under the following parameters: resolution of 120,000, scan range of 260–2,000 m/z, capillary temperature of 275°C.

Peptide identification was conducted by Proteome Discoverer software (version 2.4; Thermo Fisher Scientific) using non-deuterated samples. HDExaminer (version 3.2.1; Sierra Analytics, Modesto, CA, USA) was used to analyze the deuterium uptake level. After the manual inspection of each peptide, statistical comparisons of deuterium uptake for the control and the heated rAAV8s determined the criteria for defining a significant difference in accumulated deuterium uptake for all labeling conditions (i.e., >3.281 Da). The regions with significant differences between the two states were mapped onto the crystal structure of the AAV8 capsid (PDB code: 2QA0) and the structure of VP1 N-termini, which was predicted using LocalColabfold (version 1.5.5) owing to a lack of structural determination.^35^

### Sedimentation velocity analytical ultracentrifugation

Samples of rAAV8 with a UV absorbance of 0.571 at 230 nm were incubated on ice (control), 50°C, and 60°C for 15 min. Each incubated sample (390 μL) was loaded into a 12-mm double-sector charcoal-filled Epon centerpiece, and 390 μL of a corresponding solvent was loaded into each reference sector. The data were collected at 20°C using an Optima AUC instrument with a 50 Ti Rotor (Beckman Coulter, Brea, CA, USA) at 3,000 or 20,000 rpm with UV detection at 230 and 260 nm. After measurement, each sample was collected; mixed with 18 μL of Benzonase in 20 mM Tris-HCl, 2 mM MgCl_2_, 20 mM NaCl, and 1× PBS with 0.001% poloxamer 188; diluted with 200 mM NaCl to a total volume of 360 μL; and incubated at 37°C for 30 min. Each incubated sample was loaded into a 12-mm double-sector charcoal-filled Epon centerpiece, and the same volume of the corresponding solvent was loaded into each reference sector. The data were collected using the same method as used for Benzonase-untreated samples. The collected data were analyzed using a continuous *c*(s) distribution model. The refractive index increment (*dn/dc*) of AAV8 EPs was calculated using Sednterp software, version 3.

### Electrostatic surface potential

The structures were used as same as that used for the mapping of HDX-MS results. Before the electrostatic surface was computed, the titration state of each structure was estimated and protonated in a manner consistent with favorable hydrogen bonding at pH 7 using PDB2PQR software.^36^^,^^37^ The electrostatic surfaces of these structures were calculated using Adaptive Poisson-Boltzmann Solver software.^38^ The ionic strength was set to 0.15 M.

## Data availability

The data in the present study are available from the corresponding author upon reasonable requests.

## Acknowledgments

The present study was supported by a grant-in-aid from “Research and development of core technologies for gene and cell therapy” supported by the 10.13039/100009619Japan Agency for Medical Research and Development (AMED) (grants JP21ae0201001, JP21ae0201002, and JP24se0123004h0101). Computations were partially performed on the NIG supercomputer at ROIS National Institute of Genetics. We thank Takahiro Maruno, Takaaki Kurinomaru, and Kimitoshi Takeda (U-medico, Osaka, Japan) for the discussion with AUC and MP experiments and Hiroaki Oyama (Osaka University, Osaka, Japan) for the support of HDX-MS experiments. We also thank BASF for providing the Poloxamer 18ｖ8. We thank Edanz (https://jp.edanz.com/ac) for editing a draft of this manuscript.

## Author contributions

Conceptualization, Y.Y., S.S., and S.U.; investigation, Y.Y., S.S., and T.I.; resources, T.I., M.F., M.A.V.R., R.N., K.H., Y.T., and R.S.; visualization, Y.Y. and S.S.; writing – original draft, Y.Y. and S.U.; writing – review & editing, Y.Y., S.S., T.T., and S.U.

## Declaration of interests

The authors declare the following financial interests/personal relationships that may be considered as potential competing interests: M.F.’s relationship with U-Medico Inc. (employment), R.N.’s relationship with Shimadzu Corp. (employment), and S.U.’s relationship with U-Medico Inc. (founder, shareholder, and CSO).

## Supplemental information

Document S1. Figures S1–S10 and Equation S1

Document S2. Article plus supplemental information
