# Supplementary material for: Unfolding of viral protein 1 N-termini facilitates genome ejection from recombinant adeno-associated virus serotype 8
Source: Mol Ther Methods Clin Dev. 2025 Apr 24;33(2):101480. doi: 10.1016/j.omtm.2025.101480 (PMC12136888; doi:10.1016/j.omtm.2025.101480)
Supplement: Document S1. Figures S1–S10 and Equation S1 [file mmc1.pdf]

OMTM, Volume *33*

Supplemental information

Unfolding of viral protein 1 N-termini facilitates genome ejection from recombinant adeno-associated virus serotype 8

Yuki Yamaguchi, Saki Shimojo, Tomohiko Ikeda, Mitsuko Fukuhara, Yasuo Tsunaka, Risa Shibuya, Mark Allen Vergara Rocafort, Ryoji Nakatsuka, Kiichi Hirohata, Tetsuo Torisu, and Susumu Uchiyama

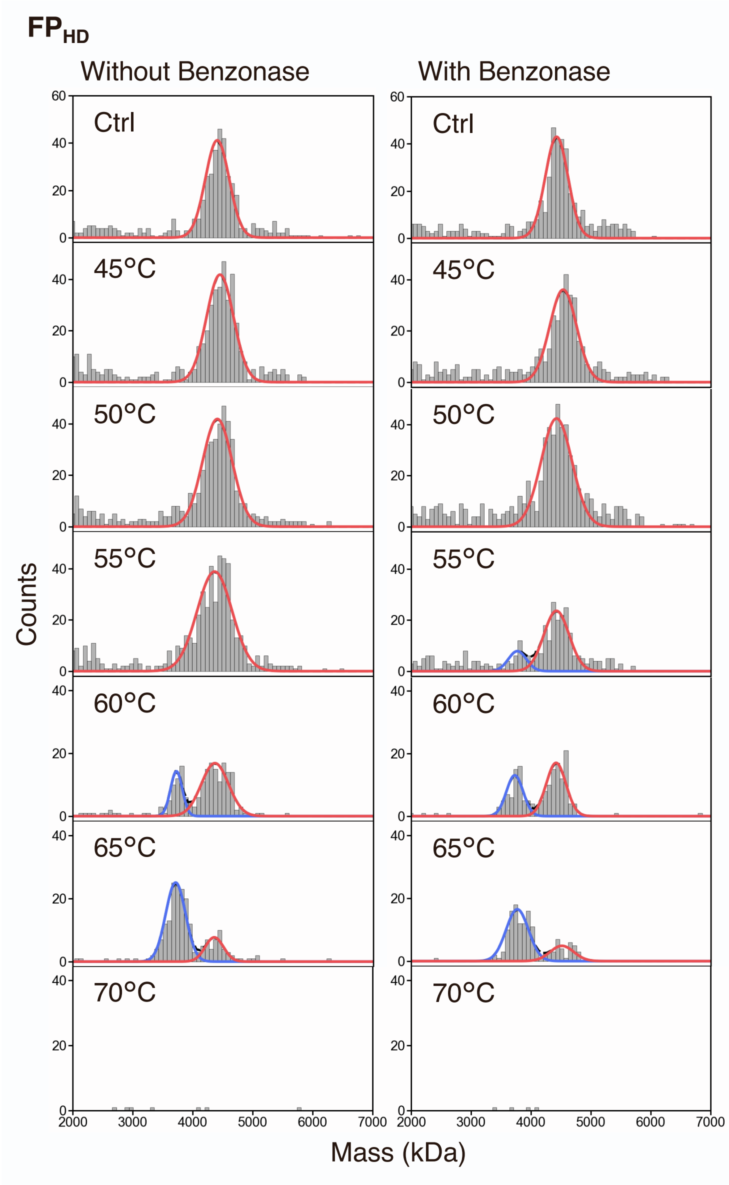

Figure S1. MP histograms of Benzonase-treated and untreated FP_{HD} after incubation on ice (control), or at 45°C, 50°C, 55°C, 60°C, 65°C, or 70°C.

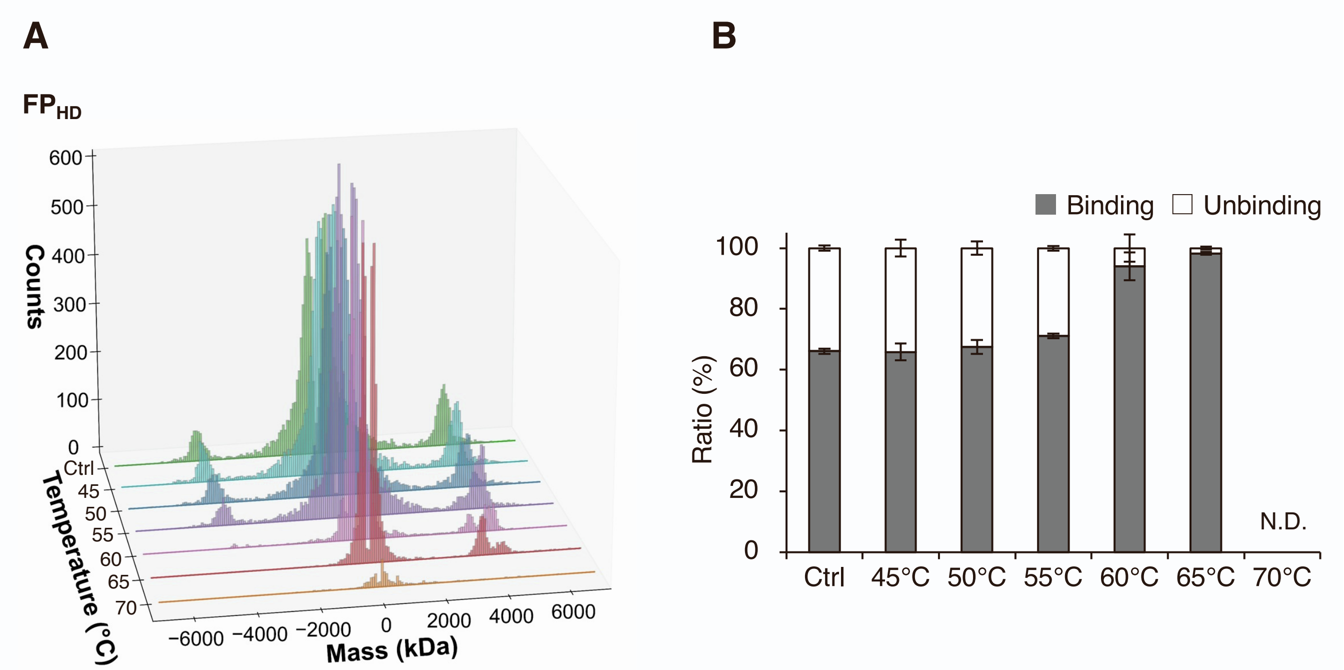

Figure S2. (A) MP histograms expanded for the unbinding side for FP$_{HD}$, and (B) the ratio of unbinding and binding particles to total particle counts of FP$_{HD}$ after incubation on ice (control), or at 45°C, 50°C, 55°C, 60°C, 65°C, or 70°C.

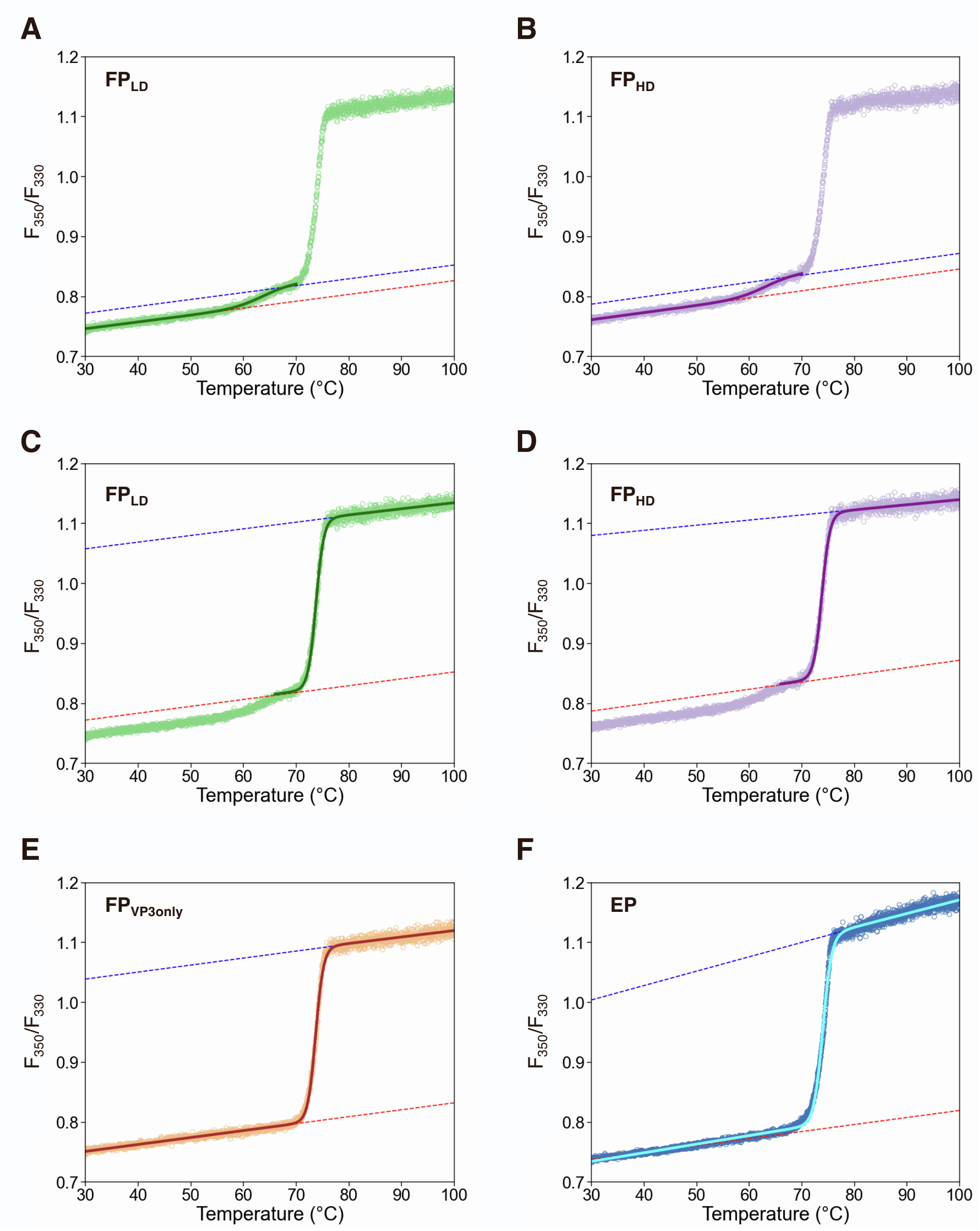

Figure S3. (A) Examples of fitting results of thermal unfolding at the first transition for FP_{LD} and (B) FP_{HD}, and at the second (major) transition for (C) FP_{LD}, (D) FP_{HD}, (E) $FP_{VP3only}$, and (F) EP.

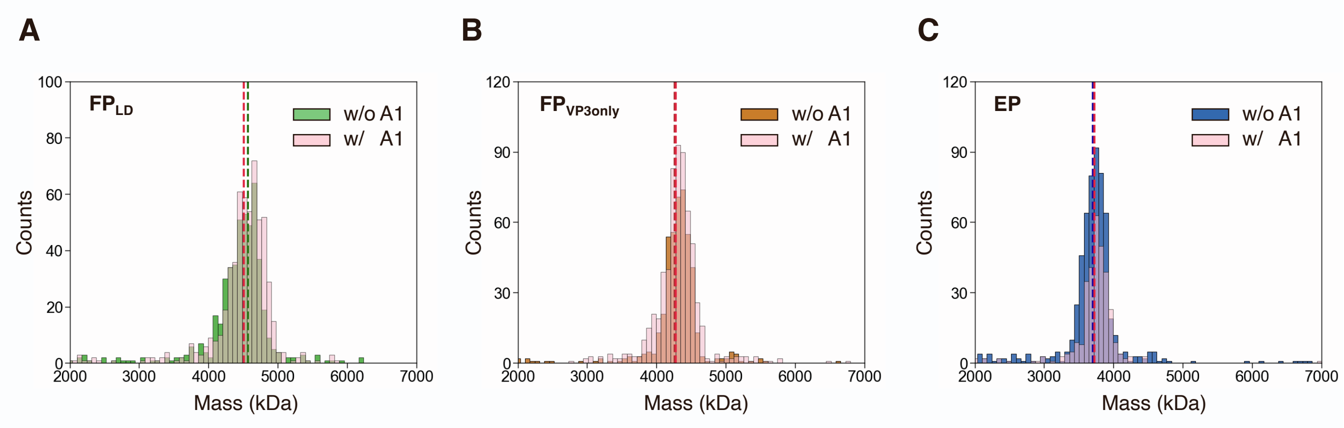

Figure S4. (A) MP histograms of FP$_{LD}$, (B) FP$_{VP3only}$, and (C) EP with and without incubation with A1 antibody before heating (control).

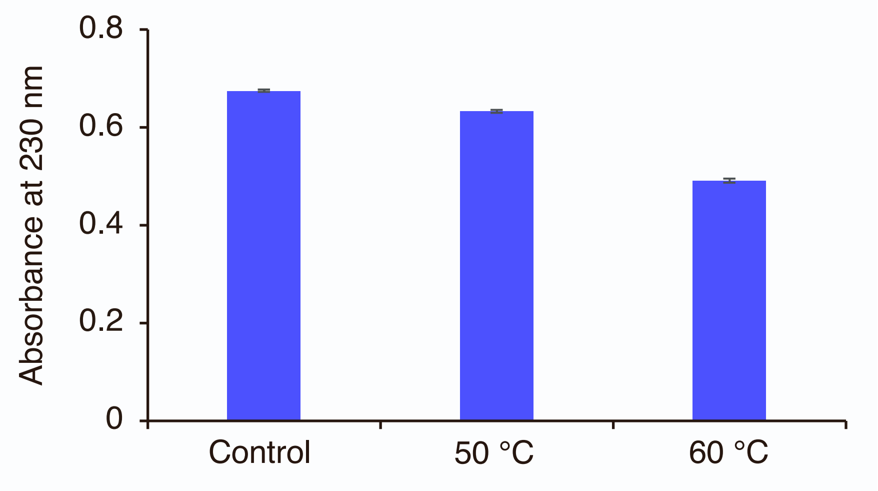

Figure S5. Average absorbance values with standard deviations at 230 nm of 6.2–6.4 cm radius for a Benzonase-untreated rAAV8 sample solution obtained at SV-AUC at a speed of 3,000 rpm.

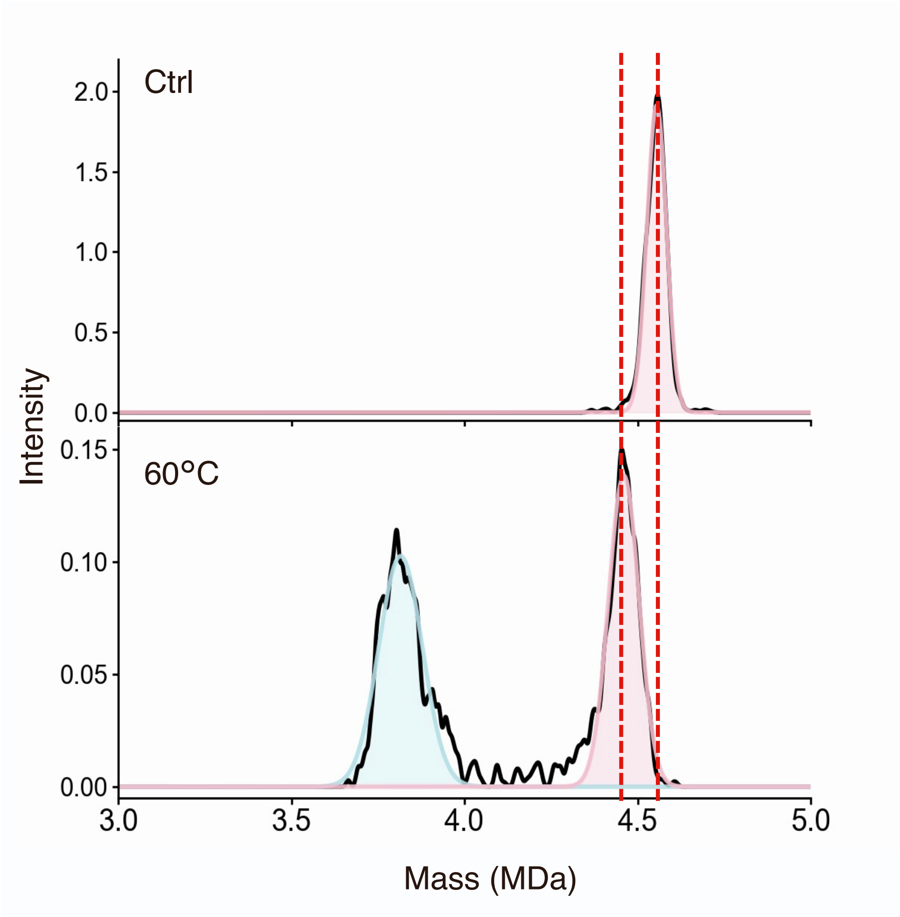

Figure S6. Orbitrap-based CD-MS distributions for the control and 60°C-heated samples.

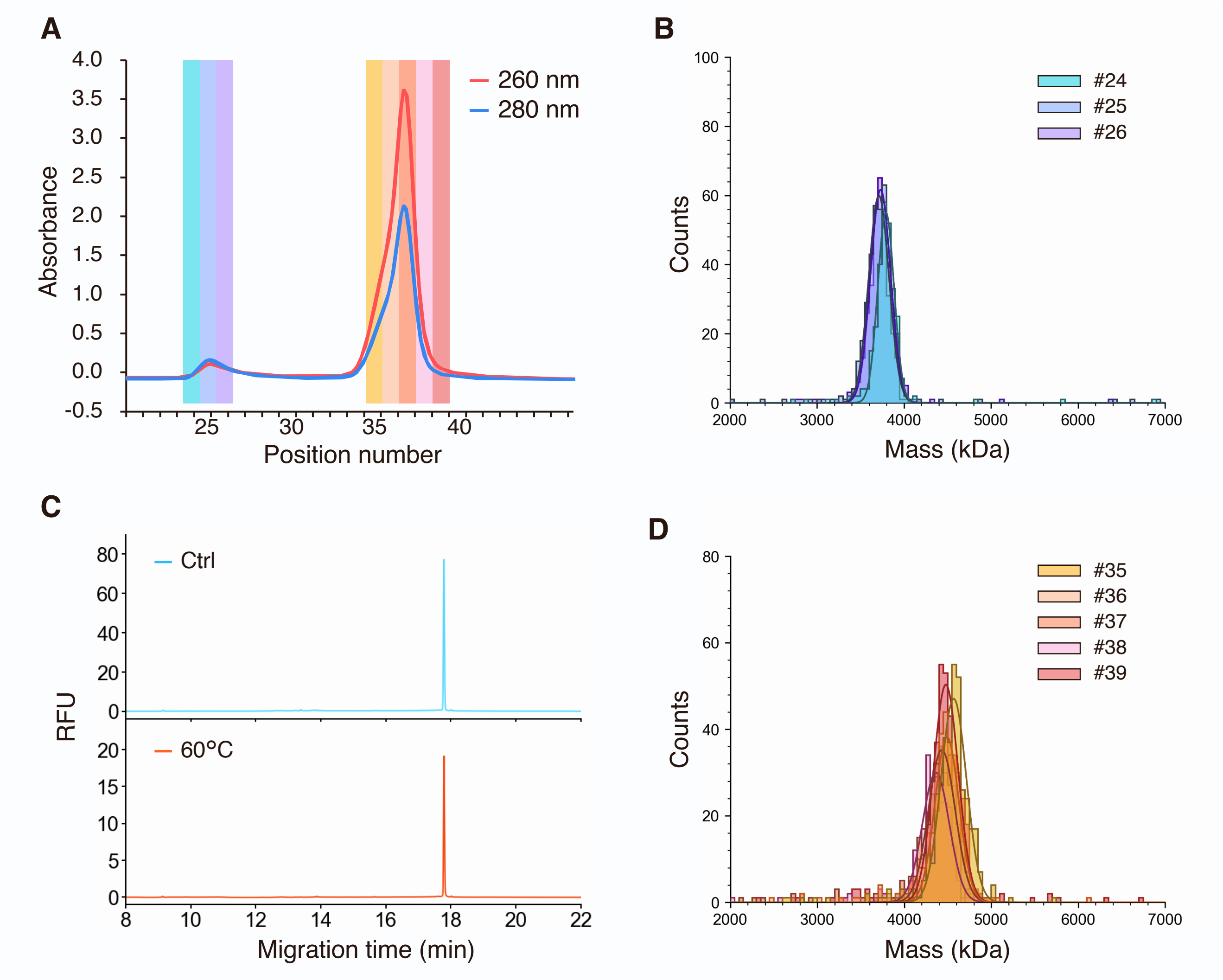

Figure S7. (A) CsCl DG-UC equilibration profiles of the rAAV8 sample after the incubation at 60°C and Benzonase treatment. The red and blue lines represent the profiles obtained at an absorbance of 260 and 280 nm, respectively. (B) MP histograms of fraction 24-26. The obtained peak corresponded to EPs. (C) CE-LIF electropherogram of ssDNA encapsidated in FPs in the control and 60°C-heated samples. (D) MP histograms of fraction 35-39. The obtained peak corresponded to FPs.

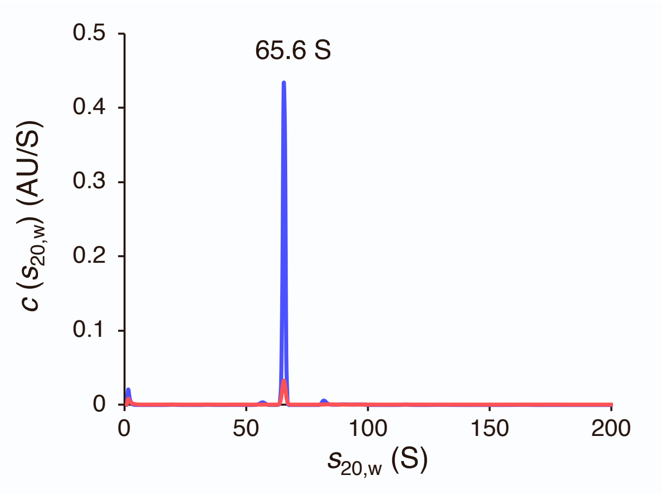

Figure S8. The sedimentation profile of highly-purified rAAV8 EP. The blue and red lines represent the profiles obtained at an absorbance of 230 and 260 nm, respectively.

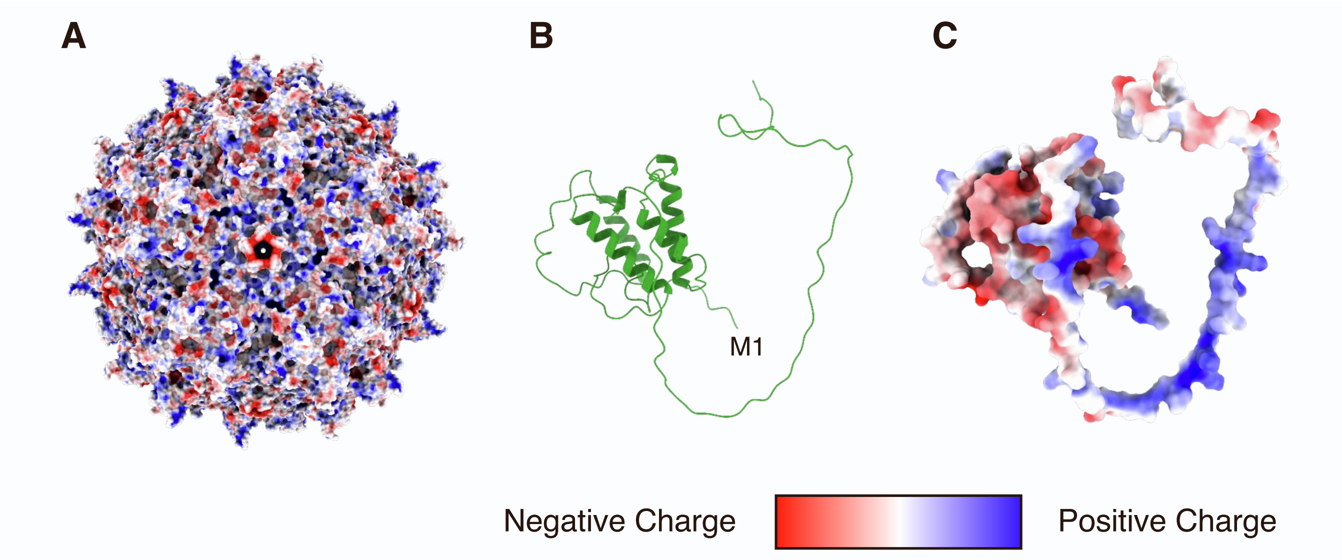

Figure S9. (A) Electrostatic surface potential model of the rAAV8 capsid. (B) Structural and (C) electrostatic surface potential models of the VP1u and VP1/VP2 common regions (VP1 N-terminus).

.

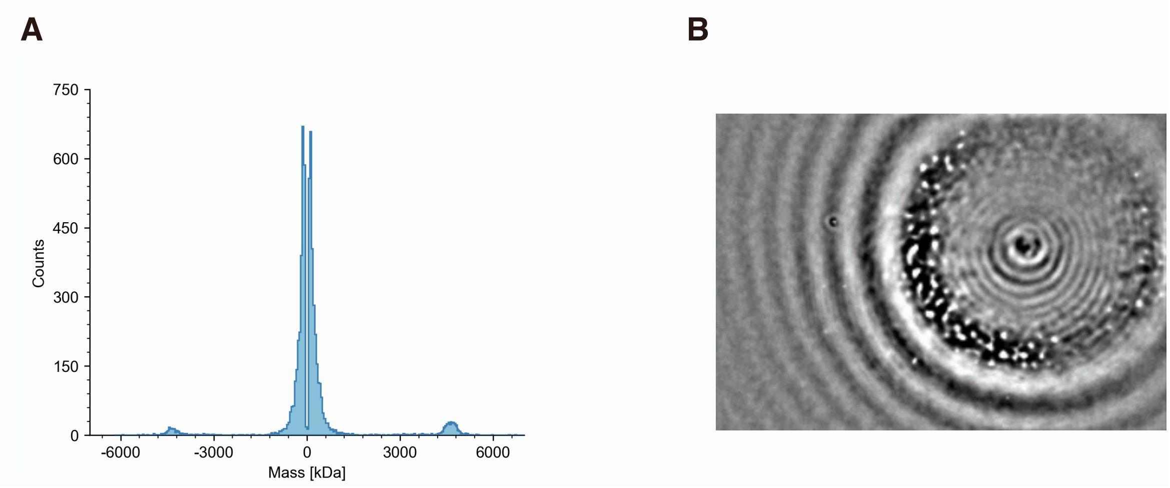

Figure S10. (A) MP histogram of rAAV8 solution which was used for SV-AUC analysis described in Figure 5A. (B) The example for rAAV8 aggregates obtained by MP.

Equation S1.

$$\boldsymbol{C\ (capsid\ particles\ (cp)/mL) = \frac{A_{230}}{\varepsilon_{230}\ (L/(mol \cdot cm))}}$$

where C is concentration of each particle, A_{230} is the peak area of each particle determined by SV-AUC measurement, and ε_{230} is molar extinction coefficient (L mol^{-1} cm^{-1}) at 230 nm of each particle.
